# Supplementary material for: Early post-partum viremia predicts long-term non-suppression of viral load in HIV-positive women on ART in Malawi: Implications for the elimination of infant transmission
Source: PLoS One. 2021 Mar 12;16(3):e0248559. doi: 10.1371/journal.pone.0248559 (PMC7954347; doi:10.1371/journal.pone.0248559)
Supplement: S1 Table — (DOCX) [file pone.0248559.s001.docx]

**S1 Table. Baseline characteristics of women who completed the study versus those lost-to-follow from study**

|  | **Completed Study  up to 24 months  of infant age** | **Lost-to-follow  from Study  But Alive on ART  at infant age 24 months** |  |
| --- | --- | --- | --- |
|  |  |  |  |
| **N** | **596** | **571** | ***p-value*** |
| **median** | 30 (25-34) | 28 (23-32) | 0.001 |
| **Mother’s age in years, n (%)** |  |  |  |
| <19 | 36 (6.0) | 34 (6.0) | 0.002 |
| 20-24 | 102 (17.1) | 144 (25.2) |  |
| 25-29 | 151 (25.3) | 153 (26.8) |  |
| > 30 | 305 (51.2) | 238 (41.7) |  |
| Missing | 2 (0.3) | 2 (0.4) |  |
| median | 3 (2-4) | 3 (2-4) |  |
| **Parity, %** |  |  |  |
| 1 | 77 (12.9) | 92 (16.1) | 0.03 |
| 2-3 | 275 (46.1) | 286 (50.1) |  |
| > 4 | 243 (40.8) | 192 (33.6) |  |
| Missing | 1 (0.2) | 1 (0.2) |  |
| **Level of Education, %** |  |  |  |
| None or primary education | 384 (64.4) | 378 (66.2) | 0.53 |
| Secondary or post-secondary education | 212 (35.6) | 193 (33.8) |  |
| **Mothers' HIV status at time of study screening, %** (4-26 weeks post-partum) |  |  |  |
| Already known HIV-infected | 579 (97.1) | 550 (96.3) | 0.43 |
| Newly diagnosed HIV-infected | 17 (2.9) | 21 (3.7) |  |
| **Mother's reported disclosure of her HIV status to her partner at enrolment** |  |  |  |
| Yes, partner knows her HIV-positive status | 501 (84.1) | 485 (84.9) | 0.73 |
| No, partner does not know her HIV-positive status | 57 (9.6) | 48 (8.4) |  |
| No partner | 33 (5.5) | 35 (6.1) |  |
| Missing | 5 (0.8) | 3 (0.5) |  |
| **Mothers' ART Initiation, %** *(r****eported at enrolment)*** |  |  |  |
| Reported to have started ART pre-conception | 288 (48.3) | 243 (42.6) | 0.07 |
| Reported to have started ART post-conception (during pregnancy or post-partum) | 278 (46.6) | 279 (48.9) |  |
| Not yet on ART (during enrolment) | 29 (4.9) | 42 (7.4) |  |
| Missing | 1 (0.2) | 7 (1.2) |  |
| **Mothers’ self-reported health status at enrolment** |  |  |  |
| Well | 560 (94.0) | 545 (95.4) | 0.21 |
| Minor Illness | 30 (5.0) | 18 (3.2) |  |
| Major Illness | 3 (0.5) | 5 (0.9) |  |
| Missing | 3 (0.5) | 3 (0.5) |  |
| **Viral Load at enrolment** |  |  |  |
| <40 | 477 (80.0) | 401 (70.2) | 0.001 |
| 40-1000 | 33 (5.5) | 53 (9.3) |  |
| >1000 | 73 (12.2) | 99 (17.3) |  |
| Unknown/Missing | 13 (2.2) | 18 (3.2) |  |
