# Supplementary material for: Early post-partum viremia predicts long-term non-suppression of viral load in HIV-positive women on ART in Malawi: Implications for the elimination of infant transmission
Source: PLoS One. 2021 Mar 12;16(3):e0248559. doi: 10.1371/journal.pone.0248559 (PMC7954347; doi:10.1371/journal.pone.0248559)
Supplement: S1 File — (PDF) [file pone.0248559.s003.pdf]

5543621376 form ID

NP01: NEMaPP SCREENING QUESTIONNAIRE

print ID [ ][ ][ ][ ][ ]

S

S01	Participant identification sticker	PLACE MOTHER STICKER HERE
S02	Date of screening	d d / m m m / 2 0 1 _ (y y y y)
S03	Staff ID	[ ][ ][ ]
S04	Mother's ANC number *(leave blank if not found)*	[ ][ ][ ][ ][ ][ ]
S05	Parity *(=number of times mother has given birth)*	[ ][ ]
S06	Mother's birthdate	d d / m m m / y y y y
S07	Age of the mother	[ ][ ] *years*
S08	Infant/child's birthdate	d d / m m m / y y y y
S09	Sex of the child	**Child1** ☐ Male ☐ Female **Child2** ☐ Male ☐ Female **Child3** ☐ Male ☐ Female
S10	Is the biological mother alive and here today?<br>Kodi ndinu kholo lomubereka mwanayu?<br>Ngati Ayi, kholo lake lili kuti?	☐ Mother is NOT alive<br>☐ Mother is ALIVE and here<br>☐ Mother is ALIVE but not here (STOP!!)
S11	Is the child less than 4 weeks of age?<br>Kodi mwanayu sanakwane masabata anayi?<br>[werengani kuchokera pa tsiku la kubadwa la mwana]	☐ No, the child is NOT younger than 4 weeks<br>☐ Yes, the child is younger than 4 weeks (STOP!!)
S12	Is the child aged 6 months or above?<br>Kodi mwanayu ali ndi miyezi isanu ndi umodzi kapena kupitilira?<br>[werengani kuchokera pa tsiku la kubadwa la mwana]	☐ No, the child is not older than 6 months<br>☐ Yes, the child is older than 6 months (STOP!!)
S13	Did you (the mother) go to ANC when you were pregnant?<br>Kodi panthawi yomwe munali oyembekezela mumapita kusikelo ya amayi oyembekezela?	☐ No<br>☐ Don't know<br>☐ Yes
S14	If YES; ANC site: [Ngati yankho ndi eya], Mumapita kuti:	[ ] × 25
S15	Were you (the mother) tested during this last pregnancy?<br>Kodi munayezedwa magazi kuti mudziwe ngati muli ndikachilombo ka HIV pamene munali oyembekezela mimba yapitayi?	☐ No, already known HIV-positive<br>☐ Not tested for HIV, other reasons<br>☐ Yes, was tested in last pregnancy

S16 What was the result of last HIV test (the mother) received during this last pregnancy?
Kodi zotsatira za kuyeza HIV zimene munalandira komaliza pa mimba yapitayi zinali zotani?
☐ Negative ☐ Not willing to reveal / don't know ☐ Positive ☐ Not tested

S17 Are you (mother or caregiver) on ART now?
Kodi mukumwa ma ARV?
☐ No ☐ Not willing to reveal ☐ Yes

S18 If Yes, when did you first start ART?
[Ngati Eya],Kodi munayamba liti kumwa ma ARV?
☐ Not applicable ☐ Before this pregnancy ☐ During 1st or 2nd trimester (1-6 mos) ☐ 3rd trimester (7+ mos) ☐ Post-partum ☐ Don't know / not willing to reveal

STAFF SAYS; We will now take a blood sample to send to the lab to test the child for HIV. We will also take a sample from you (the mother/caregiver) to test your blood now and send to the lab for confirmation. Do you have questions before we start?

Tsopano titenga magazi amwana wanu kuti titumize ku lab kuti tikayeze HIV. Titenganso magazi anu(mayi/kapena oyang'anira mwana)kuti tiyese tsopano komanso titumize ku lab kuti titsimikize ngati muli ndi kachilombo ka HIV kapena ayi. Kodi muli ndi mafunso tisanayambe?

What is the mother's (or caregiver's) rapid HIV test result today? *(follow MoH guidelines)*

S19 [DETERMINE] ☐ Negative ☐ Positive ☐ Not tested

S20 [UNIGOLD] *(if Determine is reactive)* ☐ Negative ☐ Positive* ☐ Not tested

What is the mother's (or caregiver's) rapid HIV test result on same day parallel tests ? *(if first test inconclusive)*

S21 [DETERMINE] ☐ Negative ☐ Positive ☐ Not tested

S22 [UNIGOLD] ☐ Negative ☐ Positive* ☐ Not tested

If results from same day parallel tests were inconclusive, ask the participant to return after 4 weeks.
Put the screening form in the "inconclusive" box.

What is the mother's (or caregiver's) rapid HIV test result after 4 weeks? *(if parallel testing during enrolment was inconclusive)*

S23 [DETERMINE] ☐ Negative ☐ Positive ☐ Not tested

S24 [UNIGOLD] ☐ Negative ☐ Positive* ☐ Not tested

S25 If mother's rapid test result is positive now, but reported negative during ANC in last pregnancy (S16), check ANC register or Health Passport Book for ANC HIV test result.
☐ Not applicable. *(No need to answer S26,S27)* ☐ Not found ☐ Confirmed HTC result

S26 If confirmed HTC result in ANC register or Health Passport Book, result:
☐ Negative ☐ Positive

S27 If confirmed HTC result in ANC register or Health Passport Book, date of result:
d d / m m m / y y y y

*** If rapid test (new) positive**
1. Provide post test counselling
2. Obtain consent to include mother and child in the 24 month follow up study
3. If consented to be included in the follow up study, administer enrolment form

Tell ALL mothers/caregivers that they will receive the results of the laboratory tests during the next EPI visit.
If that is not possible (i.e, if the next EPI visit is several months away, project staff will need to find a suitable time for a follow up visit. Mothers/caregivers need to be told that nobody else is allowed to collect the results on her behalf).
Interviewer says: Thank you very much for your time today

PARTICIPANT & FACILITY IDENTIFICATION [DO NOT SEPERATE PAGES OF THIS QUESTIONNAIRE]

E01 Participant identification sticker — PLACE MOTHER STICKER HERE

EA

E02 Date of enrolment — d d / m m m / 2 0 1 y (y y y y)

E03 Staff ID — ☐☐☐

E04 Infant/child's birthdate — d d / m m m / 2 0 1 y (y y y y)

E05 Sex of the child
- ☐ Male
- ☐ Female

E06 Have you (the interviewer) reviewed the biological mother's health passport? *(if possible verify the answers with health passport records)*
☐ Yes ☐ No

E07 How many times did you (the mother) go to ANC for this last pregnancy?
Munapita kangati ku sikelo ya a mayi oyembekezera ndi mimba ya mwana uyu?
☐☐ *time(s)*
☐ Don't know

E08 How many months pregnant were you (the mother) at your first visit to ANC?
Kodi sikelo munayamba muli ndi mimba ya miyezi ingati?
☐☐ *month(s)*
☐ Don't know

E09 Was the child born in a hospital or health centre?
Kodi mwanayu anabadwira kuchipatala?
- ☐ No
- ☐ Yes
- ☐ Don't know

E10 Have you (the mother) ever been tested for HIV before this last pregnancy?
Kodi munayamba mwayezetsako kachilombo ka HIV musanatenge mimba ya mwana uyu?
- ☐ No
- ☐ Yes
- ☐ Don't know

E11 For how many days did the baby get Nevirapine (ARV syrup)? (0-42 days)
Kodi mwana munamumwetsapo Neverapine (makhwala a ARV amadzi) kwa masiku angati? (Kuyambila tsiku 0-42)
☐☐ *days*
☐ Don't know

E12 Have you ever received single dose Nevirapine for prevention of HIV transmission in a previous pregnancy?
Kodi mbuyomu muli ndi mimba munayamba mwalandirako Neverapine (m'bulu omwa kamodzi mimba ikawawa) omwa kamodzi poteteza kufala kwa HIV?
- ☐ No
- ☐ Yes
- ☐ Don't know

E13 How many children has the biological mother given birth to?
Kodi munabeleka ana angati?
☐☐ *children*

E14 How many of the mothers' children have passed away?
Ndi angati omwe anamwalira?
☐☐ *children*
a + b + c + d

E15 How many of those deaths were: *(deaths should add up to answer given in E14)*
Mwa omwalira ndi angati amene :

a ☐☐ *stillbirths?* onabadwa akufa Kale
b ☐☐ *Infant deaths < 1 year?* anamwalira asanathe chaka chimodzi?
c ☐☐ *between 1-5 year deaths?* anamwalira pakati pa chaka chimodzi ndi zisanu?
d ☐☐ *> 5 year deaths?* anamwalira ndi zaka zoposera zisanu?

E16 Has the child had a DBS taken before today?
Kodi mwanayu anatengedwapo magazi (DBS) oti tidziwe ngati ali ndi kachilombo ka HIV Kapena ayi?
☐ No ☐ Yes ☐ Don't know

E17 If Yes, what was the DBS result?
[Ngati ndi eya] zotsatira zake zinatuluka bwanji?
☐ PCR negative ☐ PCR positive ☐ Results not received ☐ Not applicable

E18 If PCR positive, is the child on ART? *(not single dose neverapine prophylaxis)*
[Ngati zotsatira za magazi zinasonyeza kuti ali ndi kachilombo ka HIV] kodi mwanayu ali pa mankhwala a ARV?(osati omwa kamodzi a Nevirapine)
☐ No ☐ Yes ☐ Don't know ☐ Not applicable

E19 *(If not on ART)* Why is the child not on ART?
[Ngati mwana sali pa ARV], chifukwa chiyani sali pa ART?
☐ No test result yet ☐ Today was first positive result ☐ Other ☐ Not applicable

E20 If Other, specify:
Ngati pali chifukwa china fotokozani:

E21 *(If on ART)* Since the child's last clinic visit, how many times has the child missed ARVs?
(Ngati ali pa ART) kuchokera ulendo womaliza munabwera kuchipatala ndi mwanayu, kodi mwana anadumphitsa kumwa mankhwala masiku angati?
☐ Not on ART ☐ No doses missed ☐ 1 ☐ 2+

E22 What was the reason the child missed his/her ARVs? (check all that apply)
Chifukwa chiyani anadumphitsa kumwa mankhwala a ARV? (Chongani zonse zoyenera)
☐ Forgot ☐ Travel ☐ Sick ☐ Other ☐ Not applicable

E23 If Other, specify:
Ngati pali chifukwa china fotokozani:

NP06: NEMaPP ENROLMENT B QUESTIONNAIRE

print ID

[TO BE ADMINISTERED IN ADDITION TO THE MAIN ENROLMENT QUESTIONNAIRE]

E B

EB01 Participant identification sticker

PLACE
MOTHER STICKER HERE

EB02 What is the mother's highest level of education?
Kodi mayi munalekeza kalasi yanji kusukulu?

- ☐ none
- ☐ primary education
- ☐ secondary education
- ☐ post-secondary education

EB03 Current employment status of mother/caregiver?
Kodi mayi/oyang'anira mwana amachita chani?

- ☐ formal employment/regular job
- ☐ piece work
- ☐ own (small) business
- ☐ House wife

EB04 What is your religion?
Kodi mumapemphera chipembedzo chanji?

- ☐ Catholic
- ☐ CCAP
- ☐ Anglican
- ☐ Seventh Day Advent/Baptist
- ☐ Other Christian
- ☐ Islam
- ☐ Other
- ☐ No religion

EB05 Was this baby born at term?
Kodi mwanayu anabadwa okwanira masiku?

- ☐ no, before 38 weeks
- ☐ yes, 38-40 weeks
- ☐ after 40 weeks

EB06 What was the birth weight of your (index) baby?
Kodi mwanayu anabadwa ndi sikelo yanji?

☐ . ☐ *kgs -- (check the child's health passport book)*

EB07 Did you ever take antiretrovirals to prevent the transmission of HIV to your baby <u>in previous pregnancies</u>? (may check multiples)
Kodi munayamba mwamwako ma ARV pofuna kuteteza kupatsira HIV kwa mwana wanu pa mimba za m'mbuyomo?

- ☐ no, never had a previous pregnancy
- ☐ no, not taken antiretrovirals in previous pregnancies
- ☐ yes, Single dose NVP
- ☐ yes, several weeks of AZT around delivery
- ☐ yes, started on triple ART (1A)
- ☐ yes, started on triple ART (5A)
- ☐ don’t know

EB08 Were any of your previous children tested for HIV?:
Kodi ana ena anayezedwapo kachilombo ka HIV m'mbuyomu?

(a). How many children were tested negative?
Ndi angati sanapezeke ndi HIV?
☐☐ *children*

(b). How many children were tested positive?
Ndi angati anapezeka ndi HIV?
☐☐ *children*

(c). How many children were not tested?
Ndi angati sanayezedwe?
☐☐ *children* (REFER FOR TESTING)

1178648421 form ID

NP03: NEMaPP VISIT QUESTIONNAIRE

print ID ☐☐☐☐

PARTICIPANT & FACILITY IDENTIFICATION [DO NOT SEPERATE PAGES OF THIS QUESTIONNAIRE]

V01 Participant identification sticker — PLACE MOTHER STICKER HERE — V A

V02a Which interview is this?

- ☐ Enrolment interview ☐ Annual visit 1 at 12 months ☐ Annual visit 2 at 24 months ☐ Annual visit 3 at 36 months
- ☐ Quarterly visit 1 - Year 1 ☐ Quarterly visit 1 - Year 2 ☐ Quarterly visit 1 - Year 3 ☐ Quarterly visit 1 - Year 4
- ☐ Quarterly visit 2 - Year 1 ☐ Quarterly visit 2 - Year 2 ☐ Quarterly visit 2 - Year 3 ☐ Quarterly visit 2 - Year 4
- ☐ Quarterly visit 3 - Year 1 ☐ Quarterly visit 3 - Year 2 ☐ Quarterly visit 3 - Year 3 ☐ Quarterly visit 3 - Year 4
- ☐ Annual visit 4 at 48 months

V02b Staff ID ☐☐☐

V02c Date of visit ☐☐ (d d) ☐☐☐ (m m m) 2 0 1 ☐ (y y y y)

V03 Sex of the caregiver ☐ Male ☐ Female

V04 Are you the biological parent of the child?
Kodi ndinu kholo lomubereka mwanayu? ☐ No ☐ Yes

V05 Is the mother...
Ngati siinu mayi ake, mayi ake: ☐ Alive ☐ Dead

V06 Date of Mothers' death
Anamwalira liti? ☐☐ (d d) ☐☐☐ (m m m) 2 0 1 ☐ (y y y y)

V07 Did the mother die during child birth or within 7 days of delivery?
Kodi mayi ake anamwalira pochira kapena pasanathe masiku asanu ndi awiri atachira? ☐ No ☐ Not applicable ☐ Yes ☐ Unknown

V08 Cause of mother's death
Anamwalira ndi chiyani? ☐ Illness ☐ Not applicable ☐ Trauma/Accident ☐ Unknown

V09 How long does it take you to get to this clinic from your home?
Kodi mumatenga nthawi yaitali bwanji kuti mufike ku chipatala kuno kuchokela kwanu? ☐ < 1 hour ☐ 1-2 hours ☐ > 2 hours

V10 Do you have a spouse/partner?
Kodi muli ndi mwamuna? ☐ No ☐ Yes ☐ No answer

V11 If Yes: is he the father of this last born child?
[Ngati ndi eya] Kodi ndi bambo ake wamwanayu? ☐ No ☐ I don't have a partner ☐ Yes ☐ No answer

V12 Does your spouse / partner know your HIV status?
Kodi mwamuna wanu akudziwapo za momwe mthupi mwanu mulili zokhuzana ndi kachilombo ka HIV? ☐ No ☐ I don't have a partner ☐ Yes ☐ I am not HIV positive

V13 Has your partner ever had an HIV test?
Kodi mwamuna wanuyo anayezetsapo magazi kuti adziwe ngati ali ndi kachirombo ka HIV kapena ayi? ☐ No ☐ Don't know ☐ Yes (negative) ☐ Yes (positive) ☐ I don't have a partner

NP03: NEMaPP VISIT QUESTIONNAIRE

print ID ☐☐☐☐

V14 Are you on ART now?
Kodi muli pa mankhwala a ARV
☐ Yes (on ART now) ☐ No (never on ART) ☐ No (stopped ART)
ART #: ☐☐☐☐☐☐☐☐

V15 Have you been on another regimen before current regimen? (does not include **SdNVP)**
Kodi munayamba mwamwapo ma ARV ena musanayambe kumwa amene mukumwa panopo ?
☐ No (never before on ART) ☐ No (never other regimen) ☐ Yes (other regimen before)

V16 *(For those on ART)* How was your health when you started ART?
(Kwa amene ali pa ART) Kodi thanzi lanu linali bwanji pamene mumayamba ARV?
☐ No illness/fine ☐ A little bit sick ☐ Very sick ☐ Not applicable

V17 How do you rate your health today? Kodi thanzi lanu mukuliona bwanji lero?
☐ No illness/fine ☐ A little bit sick ☐ Very sick

V18 In the last month, how many days did you miss taking your ARVs?
Mu mwezi wapitawu, kodi mwadumphitsa kwamasiku angati osamwa mankhwala a ARV?
☐ 0 ☐ 1 day ☐ ≥2 days ☐ Not applicable

V19 What was the reason for missing your ARVs? Chifukwa chiyani munadumphitsa kumwa ma ARV?
☐ Forgot ☐ Travel ☐ Sick ☐ Other ☐ Not applicable

V20 If Other, specify:
Ngati pali chifukwa china fotokozani: ☐

V21 Have you been diagnosed with TB in the last year?
Munayamba mwapezekapo ndi chifuwa chachikulu cha TB mu chaka chapitachi? ☐ No ☐ Yes

V22 If started TB treament, start date
[Ngati ndi eya], munali pamankhwala a TB, munayamba liti?
☐☐ ☐☐☐ ☐☐☐☐
d d m m m y y y y

STAFF SAYS; Now I'm going to ask you some questions about the child here with you today. Please can I see the child's health passport? **Ogwira ntchito anene: Tsopano ndikufunsani mafunso okhudza mwana wanu muli naye lero. Chonde ndingaone nawo buku lakuchipatala la mwanayu?**

V23 Is the child sick or well today? Kodi mwanayu ali bwanji?
☐ Sick ☐ Well ☐ Died

V24 If died, date of death
[Ngati anamwalira], anamwalira liti?
☐☐ ☐☐☐ ☐☐☐☐
d d m m m y y y y

V25 How many times have you brought the child to the clinic *for a health problem* in the last three months?
(check health passport to confirm). Ndi maulendo angati amene mwabwela kuchipatala ndimwanayu akudwala pa miyezi itatu yapitayi?
☐ 0 ☐ 1 ☐ 2 ☐ 3 ☐ 4 ☐ 5+

V26 Is the child already enrolled in the HIV Care Clinic *(exposed infant clinic)?*. *Kodi mwanayu analowa kale mundondomeko ya chithandizo cha ana amene ali pachiopyezo chotenga kachilombo ka HIV?*
☐ No ---- REFER! ☐ Yes ---- HCC# ☐☐☐☐☐☐☐☐

V27 Is the child taking CPT *(Bactrim)* now? Kodi mwanayu akumwa bactrim panopa? ☐ No ☐ Yes

V28 In the last seven days, what have you fed the child? *(ask each item on the list, multiple answers possible)*
Musabata yapitawa, kodi mwana mwamudyetsa chakudya chanji?
☐ Breast milk ☐ Other milk ☐ Plumpy nut ☐ Sobo/soft drink ☐ Soup ☐ Fruit ☐ Porridge
☐ Vegetables ☐ Eggs ☐ Meat ☐ Fish ☐ Nsima/rice ☐ Other

V29 Has the child ever been admitted to hospital?
Kodi mwanayu anayamba wagonekedwapo kuchipatala?
☐ No ☐ Yes, more than once ☐ Yes, 1 time ☐ Don't know

NP08: NEMaPP Visit B QUESTIONNAIRE

print ID ☐☐☐☐

[TO BE ADMINISTERED IN ADDITION TO THE MAIN VISIT QUESTIONNAIRE]

VB01 Participant identification sticker

PLACE
MOTHER STICKER HERE

V B

VB02 which interview is this:

- ☐ Enrolment interview
- ☐ Annual visit 1 at 12 months
- ☐ Annual visit 2 at 24 months
- ☐ Annual visit 3 at 36 months
- ☐ Quarterly visit 1 - Year 1
- ☐ Quarterly visit 1 - Year 2
- ☐ Quarterly visit 1 - Year 3
- ☐ Quarterly visit 1 - Year 4
- ☐ Quarterly visit 2 - Year 1
- ☐ Quarterly visit 2 - Year 2
- ☐ Quarterly visit 2 - Year 3
- ☐ Quarterly visit 2 - Year 4
- ☐ Quarterly visit 3 - Year 1
- ☐ Quarterly visit 3 - Year 2
- ☐ Quarterly visit 3 - Year 3
- ☐ Quarterly visit 3 - Year 4
- ☐ Annual visit 4 at 48 months

VB03 Has your child been diagnosed with any of the following since the last visit :

Kodi mwana wanu anayamba wadwalako nthenda izi kuchokera pa tsiku lomaliza limene munabwela kuno?

Malaria: ☐ No ☐ Yes ☐ Don't know

Diarrhoea: ☐ No ☐ Yes ☐ Don't know

Pneumonia: ☐ No ☐ Yes ☐ Don't know

Meningitis : ☐ No ☐ Yes ☐ Don't know

TB: ☐ No ☐ Yes, on treatment ☐ Yes, not on treatment ☐ Don't know

→ TB treatment start date: ☐☐ (d d) ☐☐☐ (m m m) ☐☐☐☐ (y y y y)

Other: ☐ No ☐ Yes ☐ Don't know

QUESTION VB04 TO BE ANSWERED ONLY AT VISIT 0 (ENROLMENT) AND VISIT 1 (12MONTHS)

VB04 Has the child been immunized for hepatitis B?
(Check passport that immunization is up to date)

Kodi mwana wanu analandila katemela oteteza Hepatitis B? (yang'anani buku la mwana la sikelo)

HepB 1: ☐ No ☐ Yes

HepB 2: ☐ No ☐ Yes

HepB 3: ☐ No ☐ Yes

VB05 Please check Yes or No for each milestone: THE CHILD... Sankhani yankho loyenera pa funso lili lonse.

Smiles responsively:	☐ No	☐ Yes
Reaches/grasps objects :	☐ No	☐ Yes
Is sitting without support:	☐ No	☐ Yes
Says 3 or more words:	☐ No	☐ Yes
Picks up and eats finger food:	☐ No	☐ Yes
Is interested in other children:	☐ No	☐ Yes
Walks alone:	☐ No	☐ Yes
Feeds self with spoon (little spilling):	☐ No	☐ Yes
Combines 2 or more words:	☐ No	☐ Yes
Tries to run/can run:	☐ No	☐ Yes
Puts objects into a small container:	☐ No	☐ Yes
Uses sentences with 5 or more words:	☐ No	☐ Yes

VB06 Did you give birth since last visit? *(does not apply for enrolment visit)*

Kodi mwabeleka mwana wina kuchokela patsiku lomaliza lomwe munabwela ku kafukufuku?

☐ No -- GoTo VB16 ☐ Don't know - Go to VB16

☐ Yes ☐ No birth but abortion - Go to VB16

If YES to Question VB06 then ask questions VB07 - VB15:

VB07 Date of birth of this last baby:
kodi mwanayu anabadwa liti?
d d | m m m | y y y y

VB08 Was this last baby born at term?
Kodi mwanayu anabadwa masiku okwanira?
☐ No, before 38 weeks ☐ After 40 weeks
☐ Yes, 38-40 weeks

VB09 What was the birth outcome?
Kodi mwanayu anabadwa ndimoyo?
☐ Alive
☐ Died at #: ___ days
☐ Stillbirth

VB10 What was the birth weight of this last baby?
Kodi pamene mwanayu anabadwa analemera bwanji?
__ . __ kg ***please check health book***

VB11 How many times did you (the mother) go to ANC for this pregnancy?
Kodi munapita maulendo angati ku sikelo ya amayi oyembekezela pa mimba ya mwana uyu?
__ __ *times*

VB12 Was this child born in a health facility?
Kodi mwanayu anabadwira kuchipatala?
☐ No ☐ Yes ☐ Don't know

VB13 For how many days did this baby get Nevirapine (ARV syrup)? (0-42dy)
Kodi mwanayu analandila Neverapine(ma ARV amadzi)kwa masiku angati?
__ __ *days*

VB14 Is this recent child enrolled in the HIV Care Clinic?
Kodi mwana watsopano analowa mu ndondomeko ya ana obadwa kwa amayi amene ali ndi kachirombo ka HIV?
☐ No ☐ Yes --- HCC #:

VB15 Has this latest infant been tested for HIV?
Kodi mwana wa tsopanoyu anayezedwa kachirombo ka HIV?
☐ No [offer EID testing]
☐ Yes [provide date and result of a test below]
d d | m m m | y y y y
☐ PCR Neg
☐ PCR Pos
☐ RT Neg
☐ RT Pos

Staff Says: **We would now like to take a Blood sample from the mother (to test for viral load and CD4 count) to send to the Study Lab for testing. We will also take some measurements like height and weight of the mother and the child. These measurements will give us more information about your health. Do you have any questions before we start?**

VB16 Child's Annual Measures

Weight (kg) __ __ . __
Height (cm) __ __ __
MUAC (cm) __ __
If MUAC yellow or red, refer to nearest pediatrician or malnutrition program

VB17 MOTHERS' Annual Measures

Weight (kg) __ __ __ . __
Hip Circumference (cm) __ __ __
Waist Circumference (cm) __ __ __
MUAC (cm) __ __

VB18 Venous blood sample taken of the mother?
Kodi magazi amumtsempha a amayi atengedwa?
☐ No ☐ Yes
