# Supplementary material for: Early post-partum viremia predicts long-term non-suppression of viral load in HIV-positive women on ART in Malawi: Implications for the elimination of infant transmission
Source: PLoS One. 2021 Mar 12;16(3):e0248559. doi: 10.1371/journal.pone.0248559 (PMC7954347; doi:10.1371/journal.pone.0248559)
Supplement: Attachment — Submitted filename: Responses to Reviewers PlosONE 04Jan2021.docx [file pone.0248559.s004.docx]

PONE-D-20-36329
Early post-partum viremia predicts long-term non-suppression of viral load in HIV-positive women on ART in Malawi: Implications for the elimination of infant transmission

**Responses to reviewers’ comments:**

When submitting your revision, we need you to address these additional requirements.

1. Please ensure that your manuscript meets PLOS ONE's style requirements, including those for file naming. The PLOS ONE style templates can be found at

<https://journals.plos.org/plosone/s/file?id=wjVg/PLOSOne_formatting_sample_main_body.pdf> and

<https://journals.plos.org/plosone/s/file?id=ba62/PLOSOne_formatting_sample_title_authors_affiliations.pdf>

Response: We have verified that the manuscript meets the requirements and made a few adjustments as needed

2. Please include additional information regarding the survey or questionnaire used in the study and ensure that you have provided sufficient details that others could replicate the analyses. For instance, if you developed a questionnaire as part of this study and it is not under a copyright more restrictive than CC-BY, please include a copy, in both the original language and English, as Supporting Information, or include a citation if it has been published previously.

Response: We have included the relevant questionnaires (that include both English and the local language) as supporting information. Please note that only a selection of questions from these questionnaires were used for this sub-analysis.

3. In the Methods, please discuss whether and how the questionnaire was validated and/or pre-tested. If these did not occur, please provide the rationale for not doing so.

Response: questionnaires were pre-tested prior to study start. This has now been included in the method section.

4. In statistical methods, please refer to any post-hoc corrections to correct for multiple comparisons during your statistical analyses. If these were not performed please justify the reasons. Please refer to our statistical reporting guidelines for assistance (<https://journals.plos.org/plosone/s/submission-guidelines.#loc-statistical-reporting>).

Response: We believe that we described all the technical details and procedures required to reproduce the analysis. Post-hoc corrections were not performed/not deemed necessary. The statistical methods were reviewed and approved by 2 statisticians during CDC internal review.

5. Please include a caption for figure 2.

 Response: thanks, this has now been included

6. We note that Supporting Information S2 Data with your paper includes detailed descriptions of individual patients/participants, i.e. data of birth.

As per the PLOS ONE policy ([http://journals.plos.org/plosone/s/submission-guidelines#loc-human-subjects-research](about:blank#loc-human-subjects-research)) on papers that include identifying, or potentially identifying, information, the individual(s) or parent(s)/guardian(s) must be informed of the terms of the PLOS open-access (CC-BY) license and provide specific permission for publication of these details under the terms of this license.

Please download the [Consent Form for Publication in a PLOS Journal](about:blank) ([http://journals.plos.org/plosone/s/file?id=8ce6/plos-consent-form-english.pdf](about:blank)). The signed consent form should not be submitted with the manuscript, but should be securely filed in the individual's case notes.

Please amend the methods section and ethics statement of the manuscript to explicitly state that the patient/participant has provided consent for publication: “The individual in this manuscript has given written informed consent (as outlined in PLOS consent form) to publish these case details”.

Response: We belief that without any other data provided, participants cannot be identified through the date of birth only. However, we have taken out date of birth in the dataset. Please delete the current version from your depository.

Reviewers' comments:

Reviewer's Responses to Questions

**Comments to the Author**

1. Is the manuscript technically sound, and do the data support the conclusions?

The manuscript must describe a technically sound piece of scientific research with data that supports the conclusions. Experiments must have been conducted rigorously, with appropriate controls, replication, and sample sizes. The conclusions must be drawn appropriately based on the data presented.

Reviewer #1: Yes

Reviewer #2: Yes

2. Has the statistical analysis been performed appropriately and rigorously?

Reviewer #1: No

Reviewer #2: Yes

3. Have the authors made all data underlying the findings in their manuscript fully available?

The [PLOS Data policy](http://www.plosone.org/static/policies.action#sharing) requires authors to make all data underlying the findings described in their manuscript fully available without restriction, with rare exception (please refer to the Data Availability Statement in the manuscript PDF file). The data should be provided as part of the manuscript or its supporting information, or deposited to a public repository. For example, in addition to summary statistics, the data points behind means, medians and variance measures should be available. If there are restrictions on publicly sharing data—e.g. participant privacy or use of data from a third party—those must be specified.

Reviewer #1: Yes

Reviewer #2: Yes

4. Is the manuscript presented in an intelligible fashion and written in standard English?

PLOS ONE does not copyedit accepted manuscripts, so the language in submitted articles must be clear, correct, and unambiguous. Any typographical or grammatical errors should be corrected at revision, so please note any specific errors here.

Reviewer #1: Yes

Reviewer #2: Yes

5. Review Comments to the Author

Please use the space provided to explain your answers to the questions above. You may also include additional comments for the author, including concerns about dual publication, research ethics, or publication ethics. (Please upload your review as an attachment if it exceeds 20,000 characters)

Reviewer #1: The authors present a well described analysis of post-partum HIV viral loads among women on ART in Malawi. Overall, the analysis provides a good report from a setting that has had a recent significant scale up in viral load testing in a key population for the elimination of MTCT. I have only one major issue, which is surrounding the approach to the multivariable analysis, otherwise there are a few points that could be clarified.

Major:
Methods, lines 77. PPS sampling is implied here, but I don't think the analysis is weighted for this - this was not clear to me.

Response: Thank you, this was not correctly described and has now been amended.
PPS sampling was for the total cohort. Indeed, for the total cohort we used weighted analyses to control for the survey design. However, for this sub-set weighted analysis would not be appropriate.

Methods, lines 116-119. Stepwise selection is known to be a historically bad method of variable selection. It has many issues and does not represent a sound method of reducing/selecting variables for consideration. This is the reason that I indicated the analysis was not correct. Usually when interested in associations or risk factors we should have considered carefully the potential exposures/risk factors, as well as measured confounders, and other adjustment factors and should estimate our model from that basis. In a strictly predictive setting, again, stepwise selection would be the incorrect approach - other methods such as penalisation would perform better, but also in a strictly predictive setting we would evaluate our model at least under some sort of internal validation process (such as k-fold cross validation).

Response:

Thank you for your careful consideration of the analysis plan. We utilized the stepwise selection after discussion with CDC statisticians. After consulting again with them on receiving your review feedback, they advised that while from an academic standpoint it may not be ideal, the disadvantages are not considered ‘bad’ or ‘unsound’ as it remains a very practical approach in this context. In our early infant transmission (6-12wks of age) analysis conducted with study enrolment data, we developed and utilized a directed acyclic graph (DAG) to identify confounders and guide selection of variables (see Lancet article embedded and figure pasted below). These same variables were therefore most likely to be relevant in the model utilized at 24mos of age, and because we were able to use existing knowledge to a-priori determine what factors would be reasonable to include in the model, then the method chosen was appropriate.

If preferred, we would be happy to reference or reproduce this DAG in this paper as well, as the variables included are the same. Thank you in advance for your guidance.

Results:
The relatively small sample sizes and large OR & CI led me to question the stability of the estimates. Was any assessment for multi-collinearity and/or quasi-separation done?

Response: We agree that this final model represents a large OR and CI based on the relatively small sample size. We did not employ the methods suggested but have ensured that we are highlighting this in the limitations of our analysis.

This analysis primarily reports VLS among individuals retained - this needs to be emphasised much more in the discussion as it is very key to understanding the value of the analysis.

Response: we have emphasized this further in the discussion, as suggested.

Table 3: Proportions might be useful here, and the authors could consider a flow diagram / alluvial type plot

Response: We have tried several versions of charts but concluded that the current (sideways) flow chart is clearest. As there are several levels of comparisons, we did not complete proportions. These are described in the text.

Table 4 (and Table 5): It is unclear to me in the adjusted model if NS means not included, or not-significant. If the latter, the OR and CI intervals should be reported.

Response: Thank you – this was indeed not clear and has now been amended in tables and footnotes. “All variables were simultaneously entered in the logistic regression model as the first step and tested for removal one by one. In the multivariable analysis, only variables with significant associations in the last step are shown.”

Minor comments:
Abstract: Please report OR estimates along with CI intervals (rather than just "9 times" etc)
Response: The aORs have been added

Methods, lines 78-90: This is just not clear and I wonder if it could be rephrased so the sample size calculation for this can be better understood.
Response: this has now been rephrased.

Reviewer #2: This is a well-written paper that presents important findings on postpartum viral load outcomes in a nationally representative sample of women living with HIV in Malawi. While the study reassuringly finds higher proportions of viral suppression than reported in other settings, these still remain below global targets and this study clearly shows that women who have one raised viral load remain at risk for subsequent viremia.
Overall, the paper is very clearly presented. I have a few comments for clarity.

1. I found the wording of the first sentence of the results section in the abstract to be confusing - can you revise to be more clear that this is comparing retained in study to lost from study- were 46.5% retained and older, or just retained?
Response: this has now been made more clear

2. I appreciate that the results have been clearly presented as being among women with completed viral loads. I do think that the point raised in lines 139-141 that women who were lost from the study largely were known to be alive and on ART through routine data is important. I think the authors could make this clearer perhaps by adding this into the Figure 1 flow diagram (lost from study but known to be in care) and also in the discussion around considering the impact of missing data. I agree that viremia if anything is underestimated but the fact that most women lost from the study were still in ART care is useful to support why other missing data methods have not been used.
Response: Thank you – that is a good idea. We have now added those lost n study but know to be in care in Figure 1. The impact of missing data has also been further discussed in the Discussion

3. For the models in Table 4 and 5, it’s not clear if the variables reported as NS in the adjusted models were included in the final model but results not reported as not significant, or if these variables were kicked out of the model in the backward stepwise process. I suggest the adjusted OR and 95% CI be reported for each variable that was in included in the final model following the stepwise selection rather than reporting as NS.
Response: the aORs and 95% CI have now been added to the tables for those that were not significant but included in the model.

4. Do the authors have any thoughts on what could be done to best use the viral load information when it is available? When a woman has a raised VL postpartum she is at increased risk of either staying viremic or having subsequent viremia – what sort of interventions could be implemented at that time?
Response: We have added our suggestion to the results – that where resources are limited for VL, targeting at least one in the PP period would allow for identification of those women at highest risk of non-suppression throughout PP. (Ideally this VL would be during pregnancy as well, but this is beyond the scope/implications of our PP findings).

5. You may consider referencing the latest UNAIDS targets (in the 2020 world AIDS report) which include targets for viral suppression during breastfeeding (95% of pregnant and breastfeeding women living with HIV have suppressed viral loads). <https://www.unaids.org/sites/default/files/media_asset/prevailing-against-pandemics_en.pdf>

Response: Thank you. This has been added as suggested

6. PLOS authors have the option to publish the peer review history of their article ([what does this mean?](https://journals.plos.org/plosone/s/editorial-and-peer-review-process#loc-peer-review-history)). If published, this will include your full peer review and any attached files.


If you choose “no”, your identity will remain anonymous but your review may still be made public.


**Do you want your identity to be public for this peer review?** For information about this choice, including consent withdrawal, please see our [Privacy Policy](https://www.plos.org/privacy-policy).

Reviewer #1: No

Reviewer #2: No
