# Supplementary material for: Early post-partum viremia predicts long-term non-suppression of viral load in HIV-positive women on ART in Malawi: Implications for the elimination of infant transmission
Source: PLoS One. 2021 Mar 12;16(3):e0248559. doi: 10.1371/journal.pone.0248559 (PMC7954347; doi:10.1371/journal.pone.0248559)
Supplement: Attachment — Submitted filename: Second Response to Reviewer PlosONe .docx [file pone.0248559.s005.docx]

PONE-D-20-36329R1
Early post-partum viremia predicts long-term non-suppression of viral load in HIV-positive women on ART in Malawi : Implications for the elimination of infant transmission
PLOS ONE

Response to comment Reviewer 1:

==============================
Reviewer # 1, when preparing the second revised version, please remove from the manuscript the multivariable results, which are not relevant and actually misleading.
==============================

Thank you for your thorough review.

We consulted with academic and programmatic statisticians and discussed the feedback received.
As described in the former response to reviewers’ comments we were advised that while from an academic standpoint our analyses may not be ideal, that the disadvantages were not considered ‘bad’ or ‘unsound’ as it remains a very practical approach in this context. We also explained that in our early infant transmission (6-12wks of age) analysis conducted with study enrolment data, we developed and utilized a directed acyclic graph (DAG) to identify confounders and guide selection of variables (see Lancet article: https://www.thelancet.com/pdfs/journals/lanhiv/PIIS2352-3018(18)30316-3.pdf). These same variables were therefore most likely to be relevant in the model utilized at 24mos of age, and because we were able to use existing knowledge to a-priori determine what factors would be reasonable to include in the model, then the method chosen was appropriate.

After careful consideration, we did not remove the multivariable results from the manuscript, as we disagree that the nuanced method changes would result in a measurable difference to the findings, and the paper would ultimately be of lower quality.  As pragmatic researchers with a focus on creating evidence for programmatic improvement, we believe that our conclusions that women with non-suppressed viral load at early postpartum are likely to remain elevated at 12 months and 24 months needs to be published (ie. a single VL measure at this timepoint can identify the majority of women who remain unsuppressed).
